# Measuring parental involvement as parental actions in children’s private music lessons in China

**DOI:** 10.3389/fpsyg.2022.1061765

**Published:** 2023-01-11

**Authors:** Cancan Cui

**Affiliations:** College of Music and Dance, Guangzhou University, Guangzhou, Guangdong, China

**Keywords:** parental involvement, parents’ actions, proactivity, passivity, avoidance

## Abstract

The purpose of this study was to establish a survey instrument to measure Chinese parents’ level of actions in their children’s private music classes. I adopted Fung’s framework of change and human actions as the theoretical support for a model of parents’ level of actions. Parents of 5- to 12-year-old children (*N* = 894) from 20 different provinces in China were surveyed on their level of involvement (i.e., proactivity, passivity, and avoidance) in their children’s private music education. Seven factors were extracted from the exploratory factor analysis, which were then consolidated into a 3-factor solution. Confirmatory factor analysis indicated an adequate model fit for the data collected from the Parents’ Level of Action in Private Music Learning Scale. The results from correlation analyses revealed that (1) children’s age had a direct but weak correlation with parents’ proactivity and (2) parents’ proactivity was positively associated with children’s intention to take music lessons. The results of the repeated-measures ANOVA indicated that most Chinese parents in this study were proactively involved in their children’s private music lessons. The findings from this study are consistent with the literature. Implications and recommendations are discussed, and suggestions for future research are included.

## Introduction

Decades of educational research, concepts, and theory development show that parents have a prominent role in inspiring and reinforcing children to achieve academic success (Pomerantz et al., 2005; Amatea et al., 2006; Shatkin and Gershberg, 2007; Black, 2022). Studies have shown that children’s academic success (Hawes and Plourde, 2005; Pomerantz et al., 2005; Duan et al., 2018) is influenced by parental involvement, as they are children’s first educators in life. In the field of music education, parents’ roles are highly meaningful (McPherson, 2008; Steinberg et al., 2021). Previous research asserted that parental support and involvement produced positive effects (Huber, 2019; Yuan et al., 2021). More intuitively, providing a musical environment, supervising activities at home and investing in children’s musical learning and activities influence children’s personality, identity, communication ability, musical competence, and accomplishments (Creech and Hallam, 2003, 2011; McPherson, 2008; MacGlone et al., 2022). Due to these benefits, an increasing number of parents in both the United States (U.S.) and China are willing to send their children to music lessons, and diverse types of music learning patterns have appeared, one of which is taking private lessons. In China alone, a recent conservative estimate suggests that approximately 40 million music learners are enrolled in piano lessons (Montefiore, 2014). This number does not include learners who are taking music lessons other than piano. Attending private music lessons has become prevalent among an increasing number of learners, especially for those aiming to pursue music as a career or to achieve success in the music domain (Bai, 2021). However, due to the intensity of junior and senior high-school studies in China (Bai, 2021), only students who desire to pursue music as a career path focus more of their efforts on practicing music. These students normally started to learn piano at the same time they started formal education (Bai, 2021). Based on recent studies and the current situation in China, elementary children’s age range normally between 5 and 12 years old (Liu et al., 2016). Therefore, this study focused on parents with children at the elementary level between 5 and 12 years old.

Researchers have yet to reach an agreement on the definition of parental involvement because “despite its intuitive meaning, the operational use of parental involvement has not been clear and consistent” (Fan and Chen, 2001, p.3). Diverse explanations that encompass multiple dimensions, such as behaviors, activities, goals, beliefs, attitudes, and outcomes, can all be utilized to interpret parental involvement (Sheldon, 2002; Hoover-Dempsey and Sandler, 2005; Jeynes, 2005; Larocque et al., 2011). The definition of “parental involvement” can also be interpreted differently from parents’, students’, and teachers’ perspectives (Barge and Loges, 2003; Epstein, 2008; Colon-Leon, 2018). Parents might be involved in children’s music lessons in diverse ways depending on the situation, which itself is subject to frequent change. Dynamic situations may be affected by multiple factors, such as the children’s schedules, attitudes, preferences, or progress, which are all potential changes that can occur in music training. This way, their parents have to decide whether they are going to act in response to the changing circumstances in their children’s private music training.

### Fung’s framework of change and human actions

Fung (2018) proposed the framework of “change and human actions” to interpret individual perceptions and responses to change. In this framework, Fung (2018) described change as incurring a decision by taking on one of two options: ignore or take action. Ignorance can be interpreted as behaving without recognizing any change in the circumstances, whereas “act” refers to recognizing change and responding to it (i.e., taking action). When individuals decide to act as a reaction to change, the level of action is determined in accordance with individual conditions (Fung, 2018). Fung (2018) further pointed out that individuals must confirm that change has occurred before deciding to act and that the level of action is influenced by people’s lives, their individual situations, or the individual’s values and priorities.

Fung (2018) identified three levels of human actions in response to change: (a) proactivity, which refers to “continuous curiosity in all types of musical experiences, regardless of one’s level of familiarity with these experiences” (p. 107). Act proactively in the field of music education refers to someone being passionate with all the details in a musical activity. For instance, a student who attends a musical concert with a lot of expectation and passion. During the concert, this student takes good notes, focusing on all the details of the musician such as their expression, breathing, and gestures, while also analyzing the musical structure and texture, and seeking out for more musical experiences. With long-term proactively involved, students may reap a lot; (b) passivity, which refers to “actions [that] are taken in recognition of the changes found in the continua only to get by without any immediate adversity” (p. 105). In relation to the field of music education, “act in passivity” refers to people taking part in music activities with a low level of desire, such as taking a required class due to its credits or to satisfy graduation requirements. If there is an option to opt out of the situation, students may want to avoid taking the class. As a result, students take this class with passivity to avoid graduation complications; and (c) avoidance, which refers to an escape from a changing environment even if the individual perceives changes and accepts it. People who “act in avoidance” in taking part in music activities lack experience in music, and there is no progress that can be made during their music experience. As for the long-term effects, it is difficult for these people to move forward musically. The information included in these three options contributes to an individual’s level of action for a particular task, and consequently, each level of action brings distinct outcomes.

The three levels of response could potentially serve as three factors in a model of parental involvement in children’s private music training. Fung (2018) claimed that “if individuals are too young to make decisions regarding their musical actions, typically their parent, guardian, or caregiver does it for them” (p. 110). Fung’s (2018) change and human actions framework provides a clear structure and solid foundation for the establishment of a measure of parental involvement, with the following three factors: (a) parents act in proactivity, viewed as the actions they take in their children’s private music lessons after they perceive and accept change from their children or themselves and are willing to change proactively. More intuitively, parents acting proactively encompasses parents’ deliberate change based on their children’s change to show their respect for their children’s preferences and the changing circumstances and to ensure their children’s music learning success. By doing so, parents acting proactively may result in many benefits for their children; (b) parents act in passivity, are parents who perceive change as something they have to make for their children. After accepting change and emphasizing it, those parents will have to make a decision toward that change, but that decision emerges with low enthusiasm or that decision was not fully explored; and (c) parents act in avoidance, are parents who make the choice to not act on change even though these parents recognize and accept that change has occurred. The main difference between “act in passivity” and “act in avoidance” in parental involvement is that in the former, parents would act even though they may not want to change; the change happens as they have to do so, therefore, they act with low enthusiasm. As for the latter, the parents realize their children’s change but refuse to act on it.

In addition to the concept of “actions,” one can easily see that “change” is another key concept in the framework; a concept that emerges in daily life, including aspects of musical life for both children and parents. In this way, Fung (2018) indicated that the deeper one delves into changes, “The more one learns about changes, the better positioned one is in making decisions to promote prosperity and to avoid adversity” (p. 96). With further elaboration, for the sake of achieving prosperity, people do not only need to emphasize the result in “change,” but they also should have a clear sense of direction of where “change” might head toward. Fung (2018) claimed that “everyone must accept the inevitability of change, so life and its meaning can be situated and at the same time human actions may have an impact on the upcoming changes” (p. 95). Furthermore, a “change” can happen unexpectedly to change a subsequent situation’s direction or to replace it. No matter if it comes conspicuously or invisibly, it indeed has a noticeable change or slight modification, and in some cases, it goes unnoticed by people.

**Figure 1:**
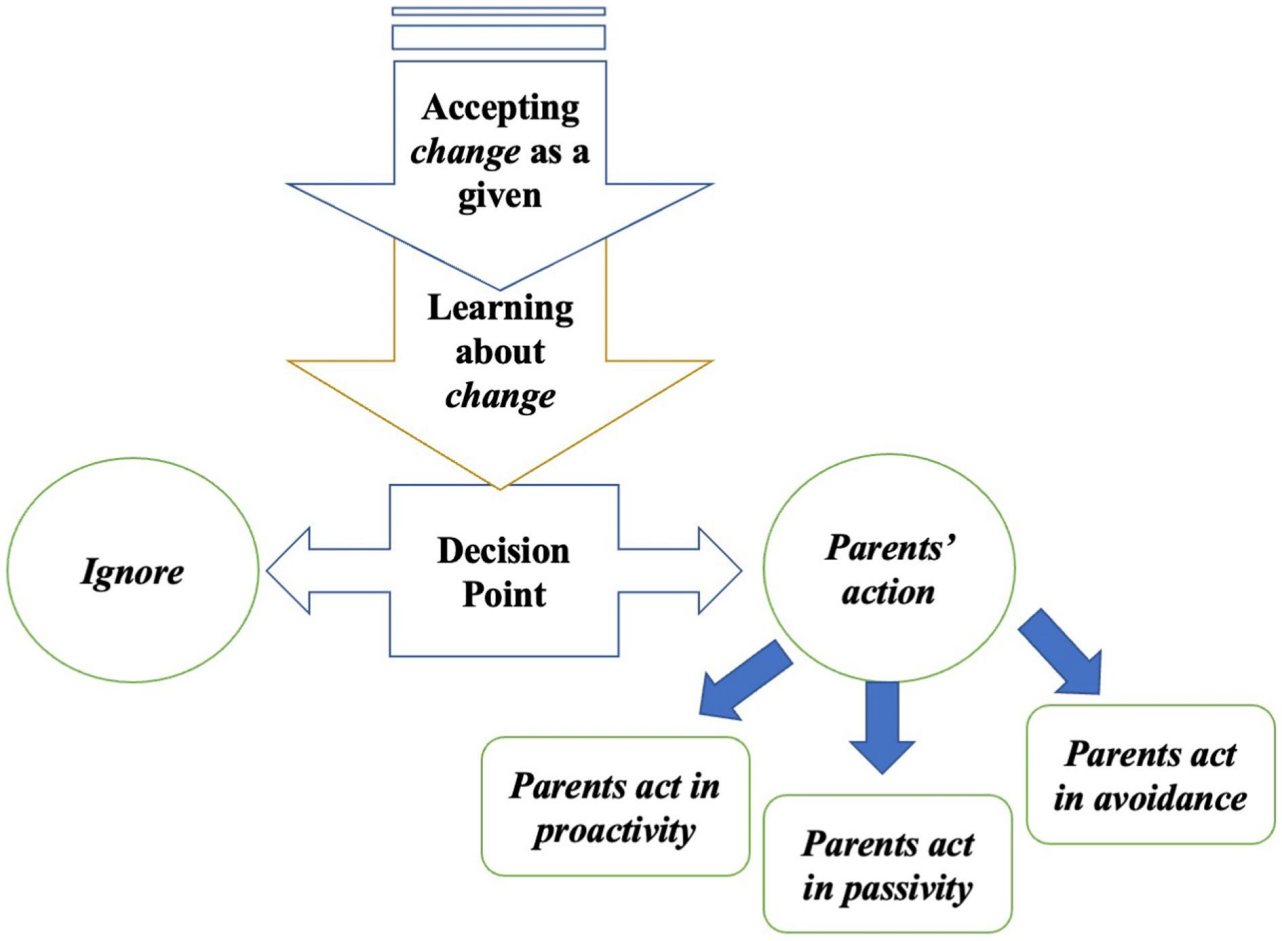
Framework of change and human actions (Fung, 2018, p. 100).

Regarding parental involvement in the field of music education, existing studies indicated that parents change based on their children’s age (Bugeja, 2009; Ho W. C., 2011). For instance, when children are still at a young age, parents are willing to spend more time supervising their children’s instrumental music practice, whereas when their children are at an older age, such as over 10 years old, parents aim to foster their independent musical abilities. These conclusions are consistent with the first two arrows leading to a decision point in Fung’s (2018) framework (see Figure 1 below). When people reach a *decision point*, people either choose to ignore the changes as if none occurred or choose to recognize the changes and “learn about the changes” before making an act actively, passively, or in avoidance.

### Purpose and research questions

Numerous research articles have acknowledged that parents’ involvement in their children’s musical development and achievement has improved in China (e.g., Ho, 2003, 2011; Shen, 2016; Upitis et al., 2017). However, there have been few investigations of the connection between parents’ level of involvement and children’s private music training outcomes (Shen, 2016; Upitis et al., 2017); in fact, as far as can be determined, studies directly and precisely examining a distinctive level of involvement in children’s private music lessons with mainland Chinese participants are rare. To date, two identified studies have examined parental involvement in some manner. For instance, Upitis et al. (2017) established a measurement instrument in parental involvement with Hong Kong parents, while Shen (2016) investigated parental involvement distinction with a small sample size between China and the United States. Hence, this study was necessary to establish an accurate understanding of parental involvement and determine whether the index established in this paper was appropriate for use with parents in mainland China. Additionally, targeted participants in this study were parents who have elementary age children between 5 and 12 years old. The 7 years of age gap between the age of 5 and 12 years old may result in considerably different outcomes. In anticipation of such differences, I intentionally examined the correlations between age and parents’ original intentions and efforts to respond to change (involvement level). These actions may imply the parents’ willingness to maintain or change their original decision to seek private music lessons. This study was guided by the following research questions:

1. What is the validity and fit index of the scale based on Fung’s (2018) framework as applied to parents with children between the ages of 5 and 12 years old who are taking private music lessons?
2. What is the reliability of this measure?
3. What are the correlations between (a) age and original intentions (children, parents, and parents’ friends and neighbors) and (b) actions responding to change (proactivity, passivity, or avoidance)?
4. What actions (proactivity, passivity, and avoidance) are parents inclined to make in their children’s private music lessons?

### Pilot study

Before the main study was conducted, I ran a pilot study with an instrument that included 98 items. These items were generated from relevant literature (McPherson, 2008; Zdzinski, 2013; Dell et al., 2014–2015), personal experiences, conversations, and life stories that were shared by parents. In the process of writing these items, the researcher initially worded items in both English and Chinese. These items were framed and organized based on the nature of parental involvement and factors that might potentially affect involvement based on the three responses to change: proactivity, passivity, and avoidance. Eighteen items were applied to investigate demographic information. Seventy-six items were related to the three choices of actions, which could be viewed as the main items of the instrument. Twenty-seven items measured whether *parents act in proactivity*, 25 items measured whether *parents act in passivity*, and another 24 measured whether *parents act in avoidance*.

Responses from a small group of participants (*n* = 33) were used to explore parents’ actions in the process of observing their children’s private music lessons and to examine the psychometric qualities of the Parents’ Level of Action in Private Music Learning Scale (PLAPMLS) with 98 initial items. Through modifications based on the IBM SPSS 27 results and the suggestions from a panel of experts in the pilot study, 58 items were retained to further explore factors that constituted the PLAPMLS (see Table 1). Nunnally (1967) recommended that the number of participants in a study should exceed a ratio of 10 participants per item when validating a measurement scale. Therefore, a large sample with diversity was necessary in this study.

**Table 1:** Number of items in pilot study and main study.

Section	Numbers of items in the pilot study	Numbers of items in the main study
Demographic questions (Part 1)	7	7
Demographic questions (Part 2)	11	11
Subtotal:	18	18
Proactivity questions	27	19
Passivity questions	25	22
Avoidance questions	24	17
Subtotal:	76	58
Preliminary items	3	3
Attention item	1	1
Subtotal	4	4
Total	98	80

In addition to 94 items in the demographic and main sections of the pilot study, three items obtained preliminary information to see if parents had already sent their children to private music lessons for at least 3 months and what their motivations were for private music education. Furthermore, the attention check item “please choose number two” was intermixed in the survey to verify whether participants carefully completed the survey and were paying attention to the questions in the questionnaire (Shamon and Berning, 2020). These made the 98 total items in the pilot study. Participants responded to each item on a 5-point Likert scale (1 = strongly disagree, and 5 = strongly agree).

## Methods

### Design and procedures

This study was descriptive in its design. Data collected from the PLAPMLS were used to construct fundamental understandings of the parental involvement as parents’ actions in children’s private music lessons and to determine if the responses from the PLAPMLS were consistent with Fung’s (2018) framework and the intended use of the scale. Data were collected using a Chinese online-based survey company (wjx.cn), a website that collects participants’ responses, stores and allocates data, and then allows for the exportation of raw data. The website provided a template that I adopted to meet the design of this study.

Purposeful snowball sampling took place in this study. The participants were recruited from across China *via* WeChat (a social media platform in China) and phone. Recruitment assistance was provided by: (1) parents whose child was taking private music lessons, (2) private music teachers who were actively teaching instruments or vocal music, and (3) general education teachers who knew of students who were taking private music lessons. Upon contact, these individuals were asked to participate in the study only if they wish to participate, and they were also asked to share the study’s recruitment with other individuals who might be interested and appeared to fit the study inclusion criteria. In this study, even though private music teachers and general education teachers were not part of the main participants group, their role and support were vital because they aided in contacting and informing their students’ parents about this study and in explaining how to participate and to distribute the online questionnaire’s link or QR code with others.

### Participants

A total of 894 parents participated in the study. The participating parents were either a father or a mother who had at least one child taking private music lessons for at least 3 months, with a variety of diverse geographic, socioeconomic, and social backgrounds. All participants were recruited from 20 provinces (Beijing, Jilin, Anhui, Shanxi, Hunan, Guangxi, Guangdong, Fujian, Liaoning, Shanghai, Inner Mongolia, Sichuan, Hubei, Henan, Jiangsu, Shandong, Zhejiang, Hebei, Chongqing, and Guizhou) across China, and the parents lived in China during the study timeframe. Although these demographic descriptors indicate diversity among the participants, the proportions of these demographic descriptors may not be an exact representation of all the parents from mainland China. Only participants with completed informed consent forms were allowed to participate in the study as required by the University of South Florida Institutional Review Board.

### Instrument

The Parents’ Level of Actions in Private Music Learning Scale (PLAPMLS) was designed and constructed to measure Chinese parents’ levels of actions in response to private musical training for their children. In this paper, I adopted Fung’s (2018) framework of change and human actions for instrument development. Within this instrument, three subscales measured three kinds of parental actions in response to their children’s private music learning, including “*parents act in proactivity*,” “*parents act in passivity*,” and “*parents act in avoidance*.” A pilot study was initially conducted with a 98-items instrument and based on the results and the Cronbach’s alpha if deleted, 18 items were eliminated from the PLAPMLS.

### Parents’ level of actions in private music learning scale

The 80-item version of the PLAPMLS used in the main study was specifically designed to measure Chinese parents’ level of involvement in private music lessons for 5- to 12-year-olds. These items were developed to reflect Fung’s (2018) three levels of action responses. Although there were 18 demographic questions, three preliminary questions, and one attention item, the remaining 58 items constituted the main questionnaire. Among these, 19 items measured parental proactivity, 22 items measured passivity, and 17 items measured avoidance (see Table 1). Participating parents rated their level of involvement in their children’s private music learning on a 5-point Likert scale (1 = strongly disagree, 2 = disagree, 3 = neutral, 4 = agree, 5 = strongly agree). The items were presented in a random order and were not grouped by source. Despite the demographic items that parents were required to answer, to ensure confidentiality, participants were not asked to provide any private information before submitting the online questionnaire. The participants spent 20 min completing the online questionnaire. Additionally, all participants remained anonymous in both the pilot study and the main study.

### Validity

To increase content validity, a bilingual music education professor and a bilingual music education doctoral student, who were fluent in both English and Chinese writing and speaking, worked on wording, framing, translating, organizing, and revising the items for almost 3 months. A backward translation was performed by another bilingual professor who was also fluent in both English and Chinese writing and speaking and assisted with the translation of the questionnaire from Chinese to English. After receiving the back-translated version, the music education professor was invited to again verify and check the entire translated questionnaire with the researcher to compare and identify the differences with the original version they had created. In the end, no major alterations were made; instead, there were a few minor modifications made to both the original English version and the Chinese version with reference to the back-translated version.

**Figure 2:**
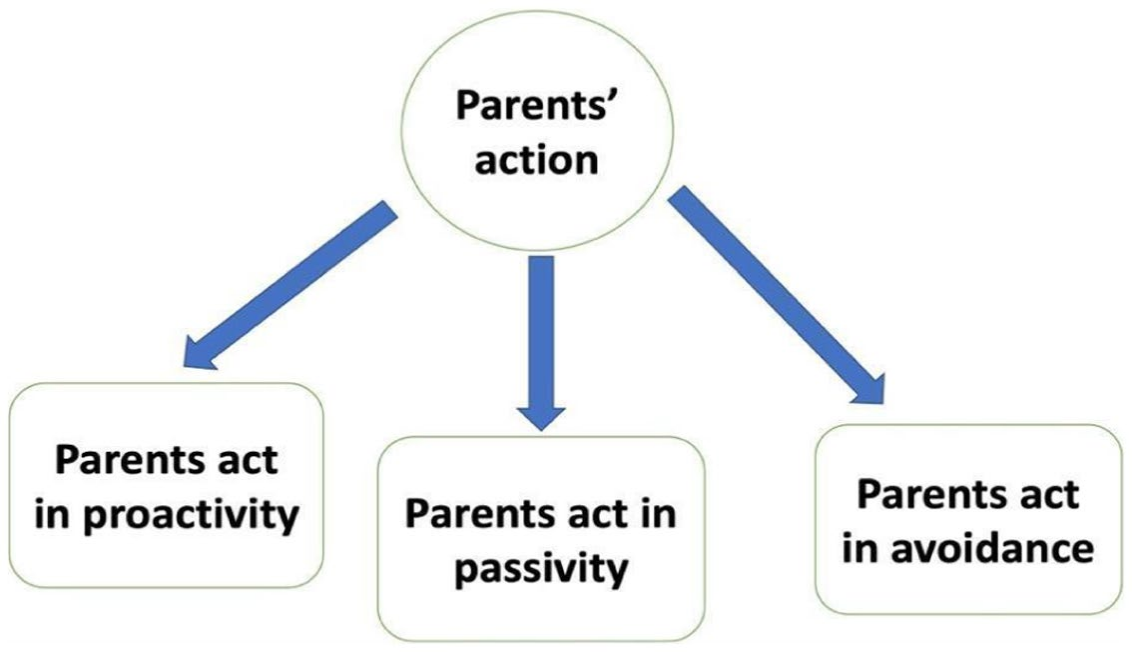
Change and human actions (adopted from Fung, 2018).

To enhance the construct validity, this study adopted Fung’s (2018) framework of change and human actions. This framework was published in *A Way of Music Education—Classical Chinese Wisdoms* in 2018. However, “change” was not the main variable in the current study; rather, this study aimed to measure how parents acted upon changes by categorizing them into three types: *proactivity*, *passivity* and *avoidance*. This study captured only a part of Fung’s (2018) change and human actions framework to establish this measurement (see Figure 2).

In addition, to enhance the item validity, I interviewed three participants who had taken part in the pilot study. Within the interview, the participants and I examined every item carefully. The participants provided specific feedback and identified the sentences or terms that could confuse respondents or contained content with ambiguous meaning. After identifying these sentences and terms, I made modifications to increase clarity and eliminate confusion.

### Demographic information

Participants were asked to report demographic information, such as parents’ gender, children’s gender, number of children, children’s age, city they were currently living in, instruments studied, length of study, length of each lesson, frequency of lessons, and tuition fees for each lesson. These questions were located separately at the beginning and at the end of the questionnaire. Seven items appeared on the first page while 11 items appeared on the last page of the questionnaire with the purpose of collecting more demographic information for this study. These initial items provided additional information that helped parents decide which child they would choose as their basis to fill out the questionnaire. All survey answers were anonymous and designed to ensure the security of personal information.

## Results

Due to the fact that the questionnaire was completed online, some participants did not complete all the items. Therefore, even though 894 participants completed the questionnaire, only 644 participants submitted valid responses. The participants were randomly split into two groups using Excel: the EFA group (*n* = 320, father = 29, mother =291; boy = 118, girl = 202) and the confirmatory factor analysis (CFA) group (*n* = 324, father = 41, mother =283; boy = 112, girl = 212).

Data from EFA are “typically used to examine common factors that explain the measured variables’ order and structure” (American Educational Research Association, American Psychological Association, and National Council on Measurement in Education, 2014). In this study, the EFA was used to verify and explore which information about the several PLAPMLS factors representing the 320 participants was consistent with Fung’s (2018) theoretical framework of change and human actions (three factors: proactivity, passivity, and avoidance). The EFA was conducted on the PLAPMLS *via* IBM SPSS version 27. The Kaiser–Meyer–Olkin (KMO) test was used to determine if the data were suitable for factor analysis (American Educational Research Association, American Psychological Association, and National Council on Measurement in Education, 2014). More intuitively, the test measures the sampling adequacy for each variable in the model. The KMO value of the PLAPMLS with 58 items was 0.91, which revealed that the sampling from the EFA was adequate and that the factor analysis may be useful with the data. Bartlett’s test of sphericity was statistically significant (*p* < 0.001), revealing that the data were likely factorable.

The results of EFA demonstrated that 12 factors were extracted, which explained 62% of the total variance for factors with eigenvalues greater than 1.00. Visual inspection of the scree plot confirmed this result. One of the factors with the lowest eigenvalues had less than three items, and cross loadings revealed that this factor was meaningless. The 12-factor solution indicated that the analysis output was inconsistent with Fung’s (2018) theoretical framework. Therefore, a decision was made to eliminate more items by adopting the factor loadings as references. Factor loadings were used to examine the relationship between the indicators and the underlying latent variable (American Educational Research Association, American Psychological Association, and National Council on Measurement in Education, 2014). Thus, items that displayed (1) low factor loading under 0.3; (2) loaded on multiple factors; or (3) loaded on only two factors but exhibited close cross loading were considered for elimination. Consequently, 33 items were eliminated.

After two rounds of item elimination, 25 items were retained in the Parents’ Level of Actions in Private Music Learning Scale (PLAPMLS). EFA was conducted again with these retained 25 items and the same 320 participants. The KMO value of the PLAPMLS with 25 items was 0.89, which was slightly lower than the 58-item value. The KMO value (0.89) was still acceptable and adequate, and Bartlett’s test of sphericity was still statistically significant (*p* < 0.001).

The EFA outputs demonstrated that seven factors were extracted, which explained 63.98% of the total variance for factors with eigenvalues greater than 1.0. As seen in Table 2, the majority of factor loadings of the indicators were greater than 0.5, thus exceeding the traditional cutoff point of factor loading of 0.40 (American Educational Research Association, American Psychological Association, and National Council on Measurement in Education, 2014). Even though there were 6 items that still displayed cross loading, these 6 items exhibited distinctive factor loadings. For instance, item 42 displayed a positive 0.60 on factor 2 and simultaneously exhibited a negative 0.3 on factor 1. However, factor 2 (avoidance) has an opposite relationship with factor 1 (proactivity; see Table 2). These opposite factor loadings supported that this item might only go under one factor. Even though these 6 items had cross loadings on two factors, the underlying latent structures of these two factors were similar.

**Table 2:** Exploratory factor analysis of PLAPMLS with varimax rotation (*n* = 320).

	Factor 1	Factor 2	Factor 3	Factor 4	Factor 5	Factor 6	Factor 7
	Proact 1	Avoid 1	Passi 1	Passi 2	Proact 2	Passi 3	Proact 3
After each private music lesson, I encourage him or her.	0.75						
After finishing each private music lesson, I reflect on it with my child together on what he or she has learned that day.	0.75						
After each private music lesson, I communicate with the music teacher about my child’s performance.	0.69						
I chat with my child regarding music often.	0.67						
After each private music lesson, I praise him or her.	0.67						
To support my child’s private music learning and provide a musical learning environment at home, I play AV materials of the performance of the instrument.	0.65						
During my child’s private music lessons, if the teacher allows, I use my phone to take video notes of my child.	0.64						
Regardless of my musical knowledge, I am willing to be part of my child’s music learning journey.	0.61						
I do not watch or listen to AV materials of the performance of the instrument with my child, even if it supports my child’s future private music learning and provides a musical learning environment at home.		0.85					
My child does not have a designated area (e.g., for storing and practicing the instruments) as his or her musical space at home, and I am not able to make it for him or her.		0.78					
I do not buy him or her musical scores, even if it may support my child’s music learning.		0.75					
I do not play AV materials of the performance of the instrument at home, even if it supports my child’s future private music learning and provides a musical learning environment at home.		0.62					
I do not purchase other musical accessories such as a metronome for my child.		0.60					
When the music teacher noticed that my child is very talented in a particular instrument and suggested learning from a more advanced music teacher, I do not take my child to the more advanced music teacher.		0.57					
Regardless of my musical knowledge, I am willing to be part of my child’s music learning journey only if my child needs me.			0.78				
I chat with my child regarding music only when he or she shows the need for it.			0.78				
To support my child’s private music learning and provide a musical learning environment at home, I play AV materials of the performance of the instrument only if needed.			0.73				
Regardless of my musical knowledge, I am willing to be part of my child’s music learning journey only if the teacher requires it.			0.65				
I enroll my child for a music level exam as other people’s children have enrolled in a music level exam.				0.80			
I purchase an instrument for my child when the price is acceptable.				0.76			
To support my child’s music learning, I buy him or her many musical books and magazines.					0.74		
In a designated area (e.g., for storing and practicing the instruments) of our home, my child has his or her own musical space.					0.72		
When I perceive that my child is gradually losing his or her interest in practicing music, I reduce the practice time.						0.83	
When my child is tired of the instrument that he or she is currently learning, I shall see if there is another instrument that is available.						0.66	
I enroll my child for an instrumental competition as I foresee its benefits for my child.							0.89

While the factor analysis results extracted seven factors from 25 items, items that loaded on one factor belonged to one subscale. According to the rotated component matrix (see Table 2), all 8 items that loaded on the first factor were under proactivity; 6 items that loaded on the second factor pertained to avoidance; items that loaded on factor 3, factor 4, and factor 6 were passivity items; items that loaded on factor 5 and factor 7 were under proactivity. Based on the outputs, items belonging to the same subscale loaded on multiple factors because parents’ actions were different depending on the age of their children or length of time spent learning music. Therefore, it was possible to combine items that pertain to the same subscale while loading on different factors together into a 3-factor solution.

Therefore, 25 items were retained (see Table 3). Among these 25 items, 11 items were under proactivity, 8 were under passivity, and 6 were under avoidance. Descriptive statistics of the remaining items were computed and are shown. Comparing the mean scores of 25 items with 58 items, the subscale “parents act in proactivity” (*M* = 3.86, SD = 0.50) still had the highest mean, and the subscale “parents act in avoidance” (*M* = 1.82, SD = 0.60) retained the lowest mean, which indicated that the pattern was consistent. Similar to the 58-item version, the kurtosis values still ranged from negative to positive (−0.31 to 0.62).

**Table 3:** Parents’ level of actions in private music learning scale (PLAPMLS; 25 items).

Subscales	Items
Parents act in proactivity	After each private music lesson, I encourage him or her. After finishing each private music lesson, I reflect on it with my child together on what he or she has learned that day. After each private music lesson, I communicate with the music teacher about my child’s performance. I chat with my child regarding music often. After each private music lesson, I praise him or her. To support my child’s private music learning and provide a musical learning environment at home, I play AV materials of the performance of the instrument. During my child’s private music lessons, if the teacher allows, I use my phone to take video notes of my child. Regardless of my musical knowledge, I am willing to be part of my child’s music learning journey. To support my child’s music learning, I buy him or her many musical books and magazines. In a designated area (e.g., for storing and practicing the instruments) of our home, my child has his or her own musical space. I enroll my child for an instrumental competition as I foresee its benefits for my child.
Parents act in passivity	Regardless of my musical knowledge, I am willing to be part of my child’s music learning journey only if my child needs me. I chat with my child regarding music only when he or she shows the need for it. I enroll my child for a music level exam as other people’s children have enrolled in a music level exam. To support my child’s private music learning and provide a musical learning environment at home, I play AV materials of the performance of the instrument only if needed. Regardless of my musical knowledge, I am willing to be part of my child’s music learning journey only if the teacher requires it. I purchase an instrument for my child when the price is acceptable. When I perceive that my child is gradually losing his/her interest in practicing music, I reduce the practice time. When my child is tired of the instrument that he/she is currently learning, I shall see if there is another instrument that is available.
Parents act in avoidance	I do not watch or listen to AV materials of the performance of the instrument with my child, even if it supports my child’s future private music learning and provides a musical learning environment at home. My child does not have a designated area (e.g., for storing and practicing the instruments) as his or her musical space at home, and I am not able to make it for him or her. I do not buy him or her musical scores even if it may support my child’s music learning. I do not play AV materials of the performance of the instrument at home, even if it supports my child’s future private music learning and provides a musical learning environment at home. I do not purchase other musical accessories such as a metronome for my child. When the music teacher noticed that my child is very talented in a particular instrument and suggested learning from a more advanced music teacher, I do not take my child to the more advanced music teacher.

Each subscale, proactivity, passivity, and avoidance, produced acceptable reliability, as determined by Cronbach’s alphas of α =0.84, α =0.77, and α =0.84, respectively. The interitem correlations ranged from 0.03 to 0.61, 0.05 to 0.52, and 0.27 to 0.70, and item-total correlations ranged from 0.20 to 0.66, 0.27 to 0.58, and 0.47 to 0.77, which were all clustered compared with the 58-item version.

The Pearson bivariate correlations of PLAPMLS with 25 items displayed negative correlations between proactivity and passivity and between proactivity and avoidance. A moderate and positive correlation between passivity and avoidance was displayed, which implied that parents provided similar interpretations of passivity and avoidance (see Table 4). Statistically significant correlations (*p* < 0.001) were still found among the three subscales.

**Table 4:** Pearson bivariate correlation of PLAPMLS (25 items) of the exploratory factor sample (*n* = 320).

	Proactivity	Passivity	Avoidance
Proactivity	-		
Passivity	−0.36***		
Avoidance	−0.46***	0.49***	-

*** indicates *p* < 0.001.

With the confirmed 25 items that constituted the PLAPMLS, a confirmatory factor analysis was needed to further ensure the accuracy of the factors and items that constituted the PLAPMLS.

### Confirmatory factor analysis results

CFA was applied to examine whether the measured variables accurately represented the number of dimensions (American Educational Research Association, American Psychological Association, and National Council on Measurement in Education, 2014). CFA helps determine whether the data output from the PLAPMLS using Fung’s (2018) theoretical framework, which represents the structural model examined, has three subscales that fit the 25-item PLAPMLS as extracted from the EFA. The results of CFA conducted with the data from 324 participants indicated that the scale did indeed measure the three levels of parental involvement (Proactivity: M = 3.84, SD = 0.48; Passive: *M* = 2.35, SD = 0.60; Avoidance: *M* = 1.84, SD = 0.64). Responses within each of the three levels of parental actions were normally distributed. Skewness values ranged from −0.01 to 0.56, and kurtosis values ranged from −0.14 to 0.00. The Cronbach’s alpha of each subscale was computed. The Cronbach’s alpha of parents acting in proactivity, parents acting in passivity, and parents acting in avoidance were α =0.83, α =0.76, and α =0.87, respectively. Each of these values was acceptable, with a value close to or greater than 0.80 (Bandalos, 2018), although one was marginally under.

Statistically significant and strong correlations between each subscale (proactivity, passivity, and avoidance) are displayed through Mplus 8.6 software. Proactivity was still negatively associated with passivity and with avoidance. Similar to the previous results, passivity was positively associated with avoidance (see Table 5). The interitem correlation ranges of each subscale (proactivity, passivity, avoidance) were from 0.04 to 0.62, 0.11 to 0.53, and 0.45 to 0.67, respectively. The item-total correlation ranges of each subscale were from 0.22 to 0.69, 0.26 to 0.59, and 0.61 to 0.77.

**Table 5:** Standardized correlation of PLAPMLS (25 items) of the confirmatory factor sample (*n* = 324).

	Proactivity	Passivity	Avoidance
Proactivity	-		
Passivity	−0.70***		
Avoidance	−0.74***	0.73***	-

*** indicates *p* < 0.001.

CFA was conducted *via* Mplus 8.6 software with three subscales and 324 participants. The items were measured in a Likert-scale format and were treated as categorical variables. Based on Hu and Bentler (1998), the cutoff points for model fit indices were comparative fit index (CFI) ≥0.90, root mean squared error of approximation (RMSEA) <0.08, and standardized root mean square residual (SRMR) <0.08. The CFA output indicated good model fit across all fit indices: CFI = 0.94; RMSEA = 0.08; SRMR = 0.06; and the Tucker–Lewis index (TLI) = 0.94, which indicated that the remaining 25 items fit well with Fung’s (2018) change and human actions framework using parents’ actions as factors.

**Figure 3:**
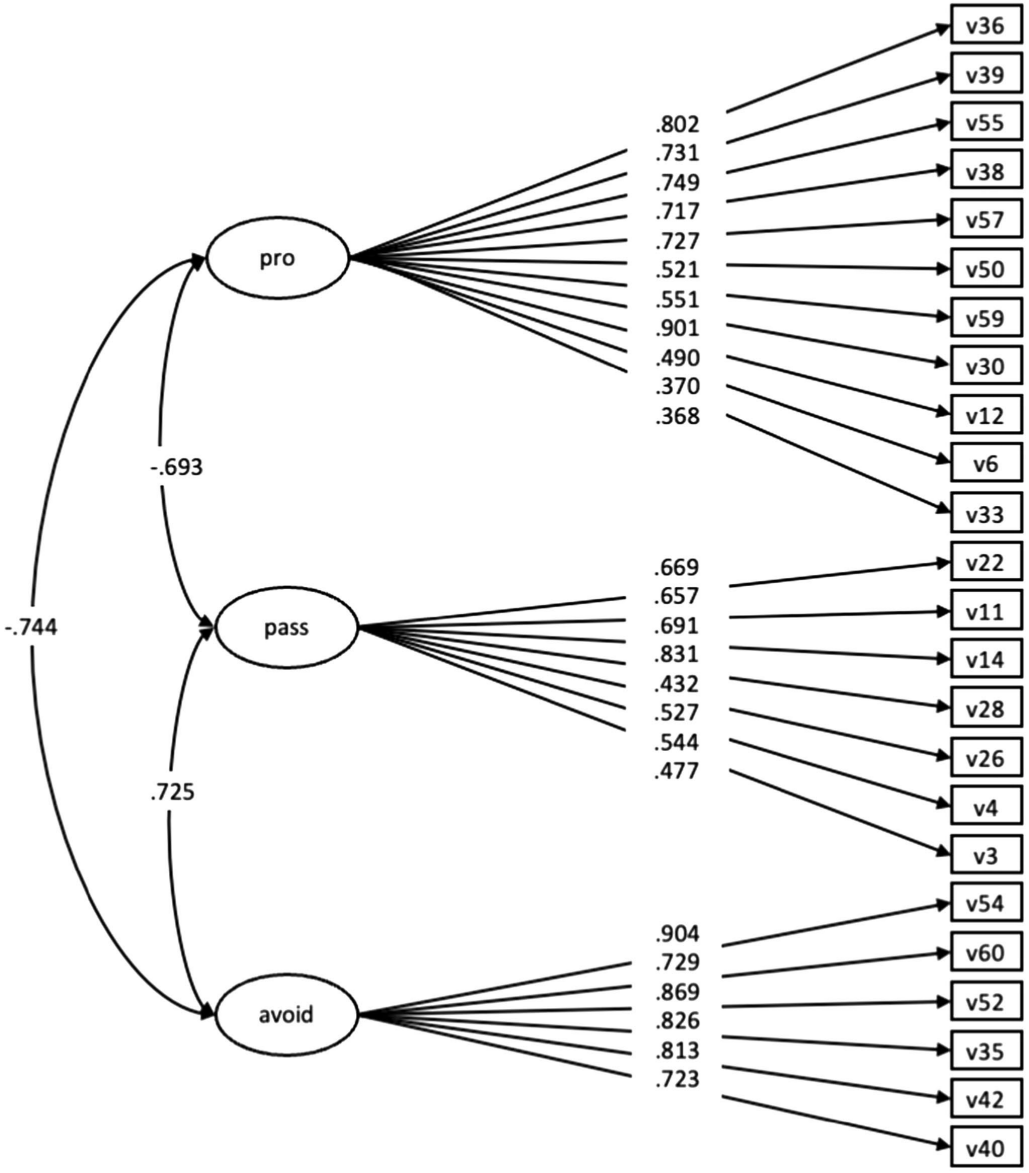
Mplus 8.6 software outputs of the confirmatory factor analysis (see Appendix).

Mplus 8.6 software automatically fixed the first item of each factor to 1.0 as reference items for model identification to obtain parameter estimates. To explicitly interpret the factor loadings, the results were reported in a standardized form. More intuitively, the standardized factor loadings of all 25 items were greater than 0.30, ranging from 0.37 to 0.90. However, Hair et al. (2014) suggested that “standardized loading estimates should be 0.5 or higher, and ideally 0.7 or higher” (p. 618). Among these 25 items, only 5 items displayed standardized factor loadings that were lower than 0.6. This result indicated that these 25 items can be examined further in the future. Examination of the measurement model analysis provided evidence that the measured variables adequately represented the three constructs (see Figure 3).

### Analysis of variance of other variables

Correlations were calculated among the children’s ages and the three levels of action (proactivity, passivity, and avoidance) to verify whether the variation in children’s age would affect parents’ actions. A statistically significant but negative correlation (*r* = −0.15, *p* < 0.01) was displayed between children’s age and parents’ proactivity (see Table 6). This result indicated that the older the children’s age was, the less proactive parents’ actions were. Additionally, children’s age seemed to have no impact on parents’ actions in either passivity or avoidance.

**Table 6:** Correlation between age and three level of actions (*n* = 324).

	Age	Proactivity	Passivity	Avoidance
Age	-			
Proactivity	−0.15**	-		
Passivity	0.07	−0.54**	-	
Avoidance	0.05	−0.57**	0.54**	-

** indicates *p* < 0.01.

In addition, there were other correlations between the original intentions (Item 1: I send my child to take private music lessons because my friend’s or my neighbor’s children are taking music lessons; Item 15: I send my child to take private music lessons because I want my child to take music lessons; and Item 62: I send my child to take private music lessons because my child wants to take it) and three levels of action (parents act in proactivity, parents act in passivity, and parents act in avoidance). Table 7 demonstrates the correlation outputs. As shown in the table, the correlation between item 1 and parents’ proactivity was statistically significant (*r* = −0.26, *p* < 0.01), which suggests that parents were less proactively involved because their original intentions were based on peer pressure. Simultaneously, statistically significant and positive correlations between preliminary item 1 and passivity (*r* = 0.31, *p* < 0.01) and between preliminary item 1 and avoidance (*r* = 0.31, *p* < 0.01) also confirmed this interpretation.

**Table 7:** Correlation between original sources of intentions and three level of actions (*n* = 324).

	1	2	3	4	5	6
Item 1	-					
Item 15	−0.10	-				
Item 62	0.18**	−0.12*	-			
Proactivity	−0.26**	−0.10	0.18**	-		
Passivity	0.31**	0.27**	−0.10	−0.54**	-	
Avoidance	0.31**	0.20**	−0.12	−0.57**	0.54**	-

*indicates *p* < 0.05, **indicates *p* < 0.01.

Item 1: I send my child to take private music lessons because my friend’s or my neighbor’s children are taking music lessons.

Item 15: I send my child to take private music lessons because I want my child to take music lessons.

Item 62: I send my child to take private music lessons because my child wants to take it.

Surprisingly, the results for the question inquiring about the correlation between item 15 “I send my child to take private music lessons because I want my child to take music lessons” and the parents’ action toward change were contradictory. In other words, the parents who originally intended to send their children to take private music lessons either responded with “passively act” or with “act with avoidance” toward their children’s music learning process. Table 7 displays statistically significant and positive correlations between both item 15 “I send my child to take private music lessons because I want my child to take music lesson” and passivity (*r* = 0.27, *p* < 0.01), and item 15 “I send my child to take private music lessons because I want my child to take music lessons” and avoidance (*r* = 0.20, *p* < 0.01). These results indicated that most parents who sent their children to take private music lessons on their own initiative were more passive or avoided involvement in their children’s music training.

The results between item 62 “I send my child to take private music lessons because my child wants to take it” and proactivity also showed that parents who acted more in proactivity tended to be those whose children’s expressed interest in music was the reason for giving them private music lessons. A statistically significant correlation was found between children’s intentions and proactivity (*r* = 0.18, *p* < 0.01; see Table 7).

A repeated-measures ANOVA was also computed to examine the effects of Chinese parents’ level of action on their children’s private music education (see Table 8). The independent variables were the three kinds of action: parents act in proactivity (*M* = 3.84, SD = 0.47), parents act in passivity (*M* = 2.35, SD = 0.60), and parents act in avoidance (*M* = 1.84, SD = 0.64) that were observed in the same group of participants. The dependent variables were the 5-point scored items. The Bonferroni pairwise comparison results (see Table 9) revealed that the highest mean score was in the category of parents acting in proactivity, which was 1.50 higher than passivity and 2.00 higher than avoidance.

**Table 8:** ANOVA results of three level of actions (*n* = 324).

	Sum of squares	df	Mean square	*F*	*p*
PLAPMLS	699.46	1, 57	446.91	936.63	0.00
Error	241.21	505.53	0.48		

**Table 9:** Mean differences of three level of actions Bonferroni pairwise comparison (*n* = 324).

Actions	Actions	Mean Differences	*p*
Parents act in proactivity	Parents act in passivity	1.50	<0.001
	Parents act in avoidance	2.00	<0.001

## Discussions and conclusions

By adopting Fung’s (2018) framework of change and human actions as a theoretical basis, this study aimed to establish the PLAPMLS to measure parents’ level of action in children’s private music learning in China and to determine whether these responses were valid and reliable representations of parents’ actions in their children’s musical education. The first and second research questions addressed the validity and reliability of the PLAPMLS instrument. Evidence from cognitive interviewing, tests of content validity, construct validity, fit indices, and reliable internal consistency indices confirmed that three subscales “parents act in proactivity,” “parents act in passivity” and “parents act in avoidance” constituted the PLAPMLS, which indicate that PLAPMLS is valid for further use as a survey instrument in measuring parents’ level of actions in children’s private music learning. With valid fit indices and reliable internal consistency, the PLAPMLS exhibited strong correlations. Noteworthy, Fung’s (2018) framework of “change and human actions” is a theoretical model that was never used to create a measurement that collected or analyzed evidence to examine the correlations between each subscale. The PLAPMLS is the first to apply this framework to create a survey instrument which approved that there is a strong correlation between each subscale under the context of parental involvement. The PLAPMLS provided solid data evidence and foundations to strengthen the theoretical framework which encourage future researchers to further explore and adapt the framework with their own data.

Results of the first and second research questions offer insights for music educators and researchers as they can gain benefits in several dimensions. On the one hand, with applying this measurement instruments to parents’ involvement, music teachers establish a collaboration with parents with a purpose of getting to know the children’s parents. More intuitively, guiding parents to assist their children’s music learning and practicing is another benefit derived from applying the instrument. In this way, music teachers and parents are able to collaborate together in order to make contributions to the students’ learning quality and achievement. On the other hand, for music researchers, this survey instrument can also serve as a measurement for parents whose children who are taking in-school music lessons or other types of musical ensembles or choir. Additionally, this instrument can be adopted and adapted to be applied on parents who have children younger than 5 or older than 12 years old. With a comparison of parents who have children within 5–12 years old, I was able to view how these parents changed their actions within this process because of their children’s age. Additionally, researchers may also discover that the influence of Fung’s framework of change and human actions may not be limited to parent’s level of actions in children’s private music learning. These same frameworks could be applied to examine the influence of the music teachers’ level of actions or music children’s level of actions.

The third research question examined the (1) correlations between children’s age and the three actions and (2) correlations between the three original motivations for music study and the parents’ three levels of action. As expected, most Chinese parents in the current study tended to reduce their involvement as their children’s age increased. This finding confirms both Bugeja (2009) and Ho W. C.’s (2011) findings that even though parents remained involved in their children’s private music education, parents who had children above 10 years old tended to reduce their involvement. This might be due to the tendency of Chinese parents to emphasize the importance of acquiring good grades in academic learning rather than musical skills (Ho H., 2011). In other words, as children near the time to prepare for the junior high school entrance test, these parents become less proactively involved in their children’s private music education.

As for the correlation analysis results between the three preliminary items and the parents’ three levels of action, they suggest that parents may be less involved because their original intentions came from peer pressure or their own motivation rather than their children’s motivation to learn music privately. However, one of the results was contradictory. “Parents who sent their children to learn music because they wanted their children to learn it” either reported “parents act in passivity” or “parents act in avoidance” in their children’s private music learning. More intuitively, parents intending to send their children to learn music but being less involved in their children’s music learning is because they believed that their children’s age allows them to learn music independently. These children are not young (i.e., pre-school age), and as such, the parents had confidence in their children’s ability to be self-regulated music learners, which prompted the parents to become less involved over time. Another possible reason behind the contradicting results might be due to the social structure of households in which both parents work and place higher priority and importance on their work within the family (Short et al., 2002). Even though these parents were anticipating being involved in their children’s music learning, they may have been stressed or occupied with their work responsibilities and as a result, they could not be as involved as they had originally anticipated. A possible way for these parents to overcome this problem is by seeking assistance from the children’s grandparents who can be proactively involved in the children’s learning process (Goh, 2009; Nyland et al., 2009).

The present study confirmed that there is a correlation between children’s intention to learn music and parents acting in proactivity. Some parents tend to be more proactively involved in their children’s private music education when the children have expressed an intention to learn music. This finding contributes to current research literature showing that most Chinese parents put their children’s interest as the dominant factor affecting the music learning process (Liu et al., 2015), rather than their own intentions or the influence of their peers. In other words, the majority of parents exhibited their concern for their children’s desires and intentions within the children’s learning journey. Both Chen et al. (2008) and Liu et al. (2015) confirmed that due to the rapid globalization process in recent years, it is possible that Chinese parents have accepted Western education and thus pay more attention to and value children’s inner intentions. Another possible reason that accords with Liu et al. (2015) is that parents whose children attend music lessons are more financially stable, and they finally take into account their children’s intentions as the dominant factor.

The fourth research question examined the effects of Chinese parents’ level of action on their children’s private music education. The results indicated that most parents in the study were proactively involved in their children’s private music education (Cho, 2015; Kong, 2021). Their proactive actions included frequently communicating with and encouraging their child, accompanying their children to their music lessons, participating in the children’s musical journey, and providing good learning resources to their children. Readers should be aware that in this study I did not attempt to recruit equal numbers of fathers and mothers. Thus, the parents who consented to participate were mostly mothers. Due to the unbalanced representation of fathers and mothers, it is unsurprising that most parents are proactively involved in their children’s private music education. This finding agrees with the conclusions from previous studies that identified the “mother” as their targeted participants (Youm, 2013; Cho, 2015; Cho and Ilari, 2021) and indicated that mothers take the dominant responsibility for caring for the children within a family or even providing “intense” support for their children (Chua, 2011; Kong, 2021), especially in China (Kong, 2021; Wang et al., 2021). Another possible reason may be due to the cultural capital (Kong, 2021). The cultural capital of Chinese parents strengthens the finding that parents are very supportive of their children’s music learning journey by providing economic support, mental support, and physical support (Kong, 2021). This study also revealed that parents use phones to record their students’ music lessons or frequently discuss music with their children, which was believed by many of the parents to stimulate students’ cognitive development and facilitate children’s musical development (Creech, 2010; Kong, 2021). Findings from the current study motivate and encourage future researchers to continue exploring the role differences between fathers and mothers.

## Future research and limitation

Future research explorations include three aspects. One direction is to continue exploring other variables that might influence parents’ level of action within their children’s private music education. In this study, only factors related to “actions” were emphasized. Even though the scale was focused on parents’ actions, some non-action factors, such as parents’ attitude/motivation or children’s learning intentions/preferences, might also affect parents’ actions toward their children’s music education.

Another direction is to explore the items in the PLAPMLS that might have correlations with musical achievement. Previous literature claimed that parental involvement plays an important role in children’s general learning process and academic learning process, and children acquire benefits from their parents’ involvement. However, there are differences between parents’ level of involvement and students’ music learning achievement. Through this study, parents can search for the most appropriate ways to be involved in their children’s music learning or even general educational learning.

Third, there is a need to explore Chinese father’s level of action in their children’s private music education (Cabrera et al., 2018). This is because the majority of parents in this study are mothers, which may have distorted the results as they do not accurately represent both Chinese parents’ (i.e., fathers’ and mothers’) actions in their children’s private music education.

This limitation of the present study should be addressed. This study, and some other studies, discovered that mothers play a dominant role within the family and have higher participation rates than fathers (Youm, 2013; Cho, 2015; Fleischmann and Haas, 2016; Jarrett and Coba-Rodriguez, 2019). To avoid inaccurate results, future researchers should recruit either fathers or mothers, or an equal amount of both, to be able to discern more accurate claims about parents’ proactivity in children’s private music learning.

## Author’s note

This article is based on the author’s dissertation, “Measuring Parental Involvement as Parental Actions in Children’s Private Music Lessons in China,” completed at the University of South Florida in 2021. I appreciated all the suggestions and feedback from my major professor C. Victor Fung, my committee members Jennifer Bugos, David A. Williams, Robert Dedrick, and professor Xuerong Cui.

## Data availability statement

The raw data supporting the conclusions of this article will be made available by the authors, without undue reservation.

## Ethics statement

The studies involving human participants were reviewed and approved by University of South Florida. The patients/participants provided their written informed consent to participate in this study.

## Author contributions

CC designed and conducted the study, completed the statistical analysis, and wrote the full manuscript. All authors contributed to the article and approved the submitted version.

## Conflict of interest

The author declares that the research was conducted in the absence of any commercial or financial relationships that could be construed as a potential conflict of interest.

## Publisher’s note

All claims expressed in this article are solely those of the authors and do not necessarily represent those of their affiliated organizations, or those of the publisher, the editors and the reviewers. Any product that may be evaluated in this article, or claim that may be made by its manufacturer, is not guaranteed or endorsed by the publisher.

## Supplementary material

The Supplementary material for this article can be found online at: https://www.frontiersin.org/articles/10.3389/fpsyg.2022.1061765/full#supplementary-material
