# Supplementary material for: Measuring parental involvement as parental actions in children’s private music lessons in China
Source: Front Psychol. 2023 Jan 11;13:1061765. doi: 10.3389/fpsyg.2022.1061765 (PMC9874687; doi:10.3389/fpsyg.2022.1061765)
Supplement: Supplementary file 1 [file Data_Sheet_1.docx]

# APPENDIX A: 98-ITEMS VERSION

**Demographic Information**

**基本信息调查：**

1. Are you a father or a mother: Father____ Mother______

您是孩子的父亲还是母亲: 父亲______ 母亲_______

1. How many children do you have? ______

您有几个孩子：______

1. How many of your children are taking music lessons? ______

您家里有几个孩子在进行课外音乐学习：________

Please select one child for the remainder of the survey if you have more than one child:

如果你有一个以上的孩子进行课外音乐学习，请选择其中一个孩子来完成以下调查：

1. Your child’s gender: Boy__ Girl___

孩子的性别是：男孩___ 女孩____

1. Your child’s age: ______

孩子的年龄是：_______

1. What is your child’s primary instrument? _______

孩子学习的主要乐器是什么：______

1. Besides the primary instrument, what other musical instrument is your child learning:______

除了以上乐器，孩子还学习其他乐器吗：________

**Please answer each one by rating yourself from 1-5, with 1 being totally disagree, to 5 totally agree.**

**请根据您的实际情况来对自己进行评估，“1”代表非常不同意，“5”代表非常同意。**

**1-Strongly disagree**

**非常不同意**

**2-Disagree**

**不同意**

**3- Neutral**

**中立**

**4- Agree**

**同意**

**5-Strongly agree**

**非常同意**

1. I send my child to take private music lessons because my friend’s or my neighbor’s children are taking music lessons. (Passivity)

1. 我送孩子去学音乐是因为我朋友或邻居的小孩都在学。

1 2 3 4 5

_______________________

1. To support my child’s music learning, I buy him/her many musical books and magazines. (proactivity)
2. 我购买很多音乐类书籍和杂志来支持孩子的音乐学习。

1 2 3 4 5

_______________________

1. During my child’s private music lessons, even if the teacher allows, I do not take any notes for my child.(avoidance)
2. 在孩子上音乐课的时候, 就算老师允许，我也不给孩子做笔记。

1 2 3 4 5

_______________________

1. To support my child’s music learning, I only buy him/her the needed musical score. (passivity)
2. 为了支持孩子的音乐学习，我只给孩子购买他需要的乐谱。

1 2 3 4 5

_______________________

1. When my child is tired of the instrument that he/she is currently learning, I change to a new instrument based on my child’s preference. (proactivity)
2. 当我的孩子厌倦了他现在学的乐器时，我遵从孩子的意愿选择了一个他喜欢的乐器。

1 2 3 4 5

_______________________

1. I purchase an instrument for my child when the price is acceptable. (passivity)
2. 当价格可接受时，我才给孩子买乐器。

1 2 3 4 5

_______________________

1. After each private music lesson, I do not praise him/her. (avoidance)
2. 每次音乐小课后，我都不表扬我的孩子。

1 2 3 4 5

_______________________

1. I enroll my child for a music level exam as I foresee its benefits for my child. (proactivity)
2. 我给我孩子报名参加考级是因为对孩子有好处。

1 2 3 4 5

_______________________

1. To support my child’s music learning, I buy him/her more musical books and magazines only if needed. (passivity)
2. 我只购买需要的音乐类书籍和杂志来支持孩子的音乐学习。

1 2 3 4 5

_______________________

1. To support my child’s private music learning and provide a musical learning environment at home, I watch or listen to AV materials in the performance of the instrument with my child together only if needed. (passivity)
2. 为了支持孩子的音乐学习以及给他在家里提供一个良好的音乐氛围，我只在需要时才跟孩子一起听看音乐会视频。

1 2 3 4 5

_______________________

1. I hire a practice supervisor for my child instead of me accompanying my child in his/her instrumental practice. (proactivity/passivity)
2. 我请了一个陪练老师来代替我陪伴孩子练习乐器。

1 2 3 4 5

_______________________

1. I enroll my child for a music level exam as other people’s children have enrolled in a music level exam. (passivity)
2. 我给我的孩子报名参加考级是因为别人的孩子都报名考级。

1 2 3 4 5

_______________________

1. If the teacher allows, I accompany my child during the private music lessons and observe. (proactivity)
2. 在孩子上音乐课的时候，如果老师允许的话，我陪在孩子旁边看他上课。

1 2 3 4 5

_______________________

1. My child does not have a designated area (e.g., for storing and practicing the instruments) as his/her musical space at home, but I am able to make it if there is extra space at home. (passivity)
2. 我孩子在家里没有固定的音乐区域（例如：练习乐器的地方，乐器摆放的地方），但是如果家里有多余的地方，我才会给孩子提供一个属于他的固定的音乐区域。

1 2 3 4 5

_______________________

1. I purchase other musical accessories, such as a metronome, for my child only if the teacher requires it. (passivity)
2. 我只给孩子购买老师要求的音乐学习辅助工具，例如：节拍器等。

1 2 3 4 5

_______________________

1. I chat with my child regarding music only when he/she shows the need for it. (passivity)
2. 当孩子有需要时，我才跟他聊音乐。

1 2 3 4 5

_______________________

1. When my child is tired of the instrument that he/she is currently learning, I communicate with my child to find out why my child dislikes it. (proactivity)
2. 当我的孩子厌倦了他现在学的乐器时，我跟孩子沟通并找出他不喜欢的原因。

1 2 3 4 5

_______________________

1. After finishing each private music lesson, I don’t reflect on it with my child. (avoidance)
2. 在孩子上完每一堂音乐小课后，我没有跟孩子一起回顾当天课上所学的内容。

1 2 3 4 5

_______________________

1. I do not take him/her to any concert and public musical activities, even if it will help my child to continue his/her music learning experience (avoidance)
2. 就算对孩子未来音乐学习有帮助，我也不带他参加任何音乐会或者其他音乐活动。

1 2 3 4 5

_______________________

1. I do not accompany his/her practice in any circumstance. (avoidance)
2. 在任何情况下我都不陪伴孩子练习乐器。

1 2 3 4 5

_______________________

1. When the music teacher noticed that my child is very talented in a particular instrument and suggested learning from a more advanced music teacher, I take my child take private music lessons even if the more advanced teacher is farther away. (Proactivity)
2. 当老师认为我的孩子在某个乐器方面很有天赋并建议孩子去跟更专业的老师学习时,就算那个老师住的很远我也送我的孩子去跟更专业的老师学习。

1 2 3 4 5

_______________________

1. When my child dislikes the music teacher with whom he/she is currently learning, I am not willing to find another teacher for him/her. (Avoidance)

22.当我的孩子不喜欢现在教他的老师时，我不会为他换老师。

1 2 3 4 5

_______________________

1. To support my child’s private music learning and provide a musical learning environment at home, I play AV materials in the performance of the instrument only if needed. (passivity)

23.为了支持孩子的音乐学习以及给他在家里提供一个良好的音乐氛围，我只在需要时才在家里给孩子播放音乐会视频。

1 2 3 4 5

_______________________

1. I send my child to take private music lessons because I want my child to take music lessons. (Proactivity)

24.我送孩子去学音乐是因为是我希望我的小孩学。

1 2 3 4 5

_______________________

1. I do not enroll my child for a music level exam. (avoidance)
2. 我不给孩子报名参加考级。

1 2 3 4 5

_______________________

1. After each private music lesson, I praise my child as needed. (passivity)
2. 每次音乐小课后，我只在需要时才表扬我的孩子。

1 2 3 4 5

_______________________

1. During my child’s private music lessons, I wait outside. (passivity)

27.在孩子上音乐课时，我在外面等。

1 2 3 4 5

_______________________

1. I purchase my child his/her own instrument to support his/her music practice. (proactivity)

28.为了支持孩子学习音乐，我给他买属于他的乐器。

_______________________

1. I do not buy my child his/her own instrument. (avoidance)
2. 我不给孩子买属于他的乐器。

1 2 3 4 5

_______________________

1. I enroll my child for an instrumental competition as other people’s children have enrolled in an instrumental competition. (passivity)
2. 我给我的孩子报名参加器乐比赛是因为别人的孩子参加了乐器比赛。

1 2 3 4 5

_______________________

1. I do not chat with my child regarding music. (avoidance)
2. 我不跟孩子聊音乐。

1 2 3 4 5

_______________________

1. When I perceive that my child is gradually losing his/her interest in practicing music, I encourage and accompany her/him to keep up with their music practice. (proactivity)
2. 当我发现孩子对练习乐器逐渐失去兴趣时，我鼓励孩子并陪伴孩子一起练习。

1 2 3 4 5

_______________________

1. I do not enroll my child for an instrumental competition. (avoidance)
2. 我不给孩子报名参加任何器乐比赛。

1 2 3 4 5

_______________________

1. Regardless of my musical knowledge, I am willing to be part of my child’s music learning journey only if my child needs me. (passivity)

34.无论我的音乐能力如何，我只有在孩子需要时才会愿意参与孩子的音乐学习。

1 2 3 4 5

_______________________

1. When the music teacher noticed that my child is very talented in a particular instrument and suggested learning from a more advanced music teacher, I take my child to the more advanced music teacher as long as it is easy to fit into our schedule (including transportation time). (passivity)

35.当老师认为我的孩子在某个乐器方面很有天赋并建议孩子去跟更专业的老师学习时,如果时间或交通方便的话，我才送孩子去跟更专业的老师学习。

1 2 3 4 5

_______________________

1. When my child is tired of the instrument that he/she is currently learning, I shall see if there is another instrument that is available. (passivity)
2. 当我的孩子厌倦了他现在所学的乐器时，我才看看是否学习其他乐器。

1 2 3 4 5

_______________________

1. After each private music lesson, I do not encourage him/her. (avoidance)
2. 每次音乐小课后，我都不鼓励我的孩子。

1 2 3 4 5

_______________________

1. Regardless of my musical knowledge, I am willing to be part of my child’s music learning journey only if the teacher requires it. (passivity)
2. 无论我的音乐能力如何，我只在老师要求时才会愿意参与孩子的音乐学习。

1 2 3 4 5

_______________________

1. I purchase other musical accessories for my child, such as a metronome. (proactivity)
2. 我给孩子购买其他音乐学习辅助工具，例如：节拍器等。

1 2 3 4 5

_______________________

1. In a designated area (e.g., for storing and practicing the instruments) of our home, my child has his/her own musical space. (proactivity)
2. 我的孩子在家里有属于他自己固定的音乐区域（例如：练习乐器的地方，乐器摆放的地方）。

1 2 3 4 5

_______________________

1. Regardless of my musical knowledge, I am willing to be part of my child’s music learning journey. (proactivity)
2. 无论我的音乐能力如何，我都会积极参与并陪伴孩子进行音乐学习。

1 2 3 4 5

_______________________

1. When my child dislikes the music teacher with whom he/she is currently learning, I am willing to change the teacher if I come across a better teacher. (Passivity)

42.当我的孩子不喜欢现在教他的老师时，如果有更好的老师出现，我才愿意为孩子换一个老师。

1 2 3 4 5

_______________________

1. To support my child to continue his/her music learning experience, I take my child to participate in more music concerts or public musical activities. (proactivity)

43.为了支持孩子继续音乐学习，我带他参加音乐会及其他音乐活动。

1 2 3 4 5

_______________________

1. When I perceive that my child is gradually losing his/her interest in practicing music, I reduce the practice time. (passivity)

44.当我发现孩子对练习乐器逐渐失去兴趣时，我减少孩子练习的次数。

1 2 3 4 5

_______________________

1. Before my child’s private music lessons, I drop my child off and leave. (avoidance)
2. 在孩子上音乐课之前，我把孩子送到上课地点就离开。

1 2 3 4 5

_______________________

1. When my child is tired of the instrument that he/she is currently learning, I do not allow my child to change to another instrument. (Avoidance)
2. 当我的孩子厌倦了他现在所学的乐器时，我也不允许他换别的乐器.

1 2 3 4 5

_______________________

1. I enroll my child for an instrumental competition as I foresee its benefits for my child. (proactivity)
2. 我给我孩子报名参加乐器比赛是因为对孩子有好处。

1 2 3 4 5

_______________________

1. I do not play AV materials in the performance of the instrument at home, even if it supports my child’s future private music learning and provides a musical learning environment at home. (avoidance)
2. 就算对孩子未来的音乐学习有帮助以及给他在家里提供一个良好的音乐氛围，我也不给孩子播放音乐会视频。

1 2 3 4 5

_______________________

1. Regardless of my musical knowledge, I am not willing to be part of my child’s music learning journey at all. (avoidance)
2. 无论我的音乐能力如何，我都不愿意参与孩子的音乐学习。

1 2 3 4 5

_______________________

1. After each private music lesson, I do not communicate with the music teacher about my child’s performance. (avoidance)
2. 每次音乐小课后，我都不询问老师孩子在课上的表现。

1 2 3 4 5

_______________________

1. After each private music lesson, I communicate with the music teacher about my child’s performance. (proactivity)
2. 每次音乐小课后，我都询问老师我孩子在课上的表现。

1 2 3 4 5

_______________________

1. After finishing each private music lesson, I reflect on it with my child together on what he/she has learned that day. (proactivity)
2. 在孩子上完每一堂音乐小课后，我跟孩子一起回顾当天在课上所学的内容。

1 2 3 4 5

_______________________

1. After each private music lesson, I encourage him/her. (proactivity)
2. 每次音乐小课后，我都鼓励我的孩子。

1 2 3 4 5

_______________________

1. When the music teacher noticed that my child is very talented in a particular instrument and suggested learning from a more advanced music teacher, I do not take my child to the more advanced music teacher. (avoidance)
2. 当老师认为我的孩子在某个乐器方面很有天赋并建议孩子去跟更专业的老师学习时,我也不送我的孩子去跟随更专业的老师学习。

1 2 3 4 5

_______________________

1. I sacrifice my free time to accompany my child in his/her instrumental practice. (Proactivity)
2. 我牺牲我的下班及休息时间陪伴孩子练习乐器。

1 2 3 4 5

_______________________

1. I do not purchase other musical accessories such as a metronome for my child. (avoidance)
2. 我没有给孩子购买其他音乐学习辅助工具，例如：节拍器等。

1 2 3 4 5

_______________________

1. Regardless of my child’s enjoyment in learning the instrument that he/she is currently learning, I decide what instrument he/she will continue to learn without communicating with my child. (avoidance)
2. 不管孩子是否喜欢他现在所学的乐器与否，我都不跟孩子沟通并自己为孩子继续学习音乐而做决定。

1 2 3 4 5

_______________________

1. I do not buy him or her other musical books or magazines even if it may support my child’s music learning. (avoidance)
2. 就算对孩子的音乐学习有帮助，我也不给孩子购买其他音乐类书籍或杂志。

1 2 3 4 5

_______________________

1. I listen to their practice while doing my daily chores and activities. (passivity)
2. 我一边做自己的事情，一边听着我孩子练习乐器。

1 2 3 4 5

_______________________

1. After each private music lesson, I communicate with the music teacher about my child’s performance only when the teacher contacts me. (passivity)
2. 每次音乐小课后，当老师先来跟我说，我才会跟老师交流我孩子在课上的表现。

1 2 3 4 5

_______________________

1. When I perceive that my child is gradually losing his/her interest in practicing music, I allow the child not to practice anymore. (avoidance)
2. 当我发现孩子对练习乐器逐渐失去兴趣时，我允许孩子不练习了。

1 2 3 4 5

____________________

1. During my child’s private music lessons, I take notes of the lesson only if the teacher requires it. (passivity)
2. 在孩子上音乐课的时候，如果老师要求，我才为他做笔记。

1 2 3 4 5

_______________________

1. After each private music lesson, I praise him/her. (proactivity)
2. 每次音乐小课后，我都表扬我的孩子。

1 2 3 4 5

_______________________

1. To support my child to continue his/her music learning experience, I take him/her to concerts or public musical activities only if the teacher requires it. (passivity)
2. 为了支持孩子继续音乐学习，如果老师要求，我才带他参加音乐会及其他音乐活动。

1 2 3 4 5

_______________________

1. I do not buy him/her musical score even if it may support my child’s music learning. (avoidance)
2. 就算对他的音乐学习有帮助我都不给孩子购买乐谱。

1 2 3 4 5

_______________________

1. When I perceive that my child is gradually losing his/her interest in practicing music, I do not allow my child to stop practicing. (proactivity)
2. 当我发现孩子对练习乐器逐渐失去兴趣时，我也不允许他不练习了。

1 2 3 4 5

____________________

1. My child does not have a designated area (e.g., for storing and practicing the instruments) as his/her musical space at home, but I am able to make it if his/her peers have it. (passivity)
2. 我孩子在家里没有固定的音乐区域（例如：练习乐器的地方，乐器摆放的地方），但是如果别的孩子都有自己固定的音乐区域，我才会为我的孩子提供。

1 2 3 4 5

_______________________

1. After each private music lesson, I encourage my child as needed. (passivity)
2. 每次音乐小课后，我只在需要的时候才鼓励我的孩子。

1 2 3 4 5

_______________________

1. I do not watch or listen to AV materials in the performance of the instrument with my child, even if it supports my child’s future private music learning and provides a musical learning environment at home. (avoidance)
2. 就算对孩子未来的音乐学习有帮助以及给他在家里提供一个良好的音乐氛围, 我也不跟孩子一起听看音乐会视频。

1 2 3 4 5

_______________________

1. When my child dislikes the music teacher with whom he/she is currently learning, I take him/her to try out more music teachers to search for the most appropriate one for him/her. (Proactivity)
2. 当我的孩子不喜欢现在教他的老师时，为了找到最适合我孩子的老师，我带着孩子去试了不同老师的课。

1 2 3 4 5

_______________________

1. During my child’s private music lessons, if the teacher allows, I take notes of the lesson by hand (proactivity)
2. 在孩子上音乐课的时候，如果老师允许的话，我手动给孩子记笔记。

1 2 3 4 5

_______________________

1. To support my child’s music learning, I buy him/her many musical scores. (proactivity)
2. 为了支持孩子的音乐学习，我给他购买很多的乐谱。

1 2 3 4 5

_______________________

1. I chat with my child regarding music often. (proactivity)
2. 我经常跟我的孩子聊音乐。

1 2 3 4 5

_______________________

1. To support my child’s private music learning and provide a musical learning environment at home, I play AV materials in the performance of the instrument. (proactivity)
2. 为了支持孩子的音乐学习以及给他在家里提供一个良好的音乐氛围，我在家播放音乐会视频。

1 2 3 4 5

_______________________

1. After finishing each private music lesson, I reflect on it only when the teacher requires me to do so. (passivity)
2. 在孩子上完每一堂音乐小课后，如果老师要求，我才跟孩子一起回顾当天课上所学的内容。

1 2 3 4 5

_______________________

1. Please choose number 2 below:
2. 请在以下数字中选择数字2：

1 2 3 4 5

_______________________

1. During my child’s private music lessons, if the teacher allows, I use my phone to take video notes of my child (proactivity).
2. 在孩子上音乐课的时候，如果老师允许的话，我通过手机录像来为他做视频笔记。

1 2 3 4 5

_______________________

1. My child does not have a designated area (e.g., for storing and practicing the instruments) as his/her musical space at home, and I am not able to make it for him/her. (Avoidance)
2. 我孩子在家里没有固定的音乐区域（例如：练习乐器的地方，乐器摆放的地方），并且我也不会为孩子在家里提供一个属于他的固定的音乐区域。

1 2 3 4 5

_______________________

1. To support my child’s private music learning and provide a musical learning environment at home, I watch or listen to AV materials in the performance of the instrument with my child. (proactivity)
2. 为了支持孩子的音乐学习以及给他在家里提供一个良好的音乐氛围，我跟孩子一起听看音乐会视频。

1 2 3 4 5

_______________________

1. I send my child to take private music lessons because my child wants to take it.
2. 我送孩子去学音乐是因为我的孩子想学。

1 2 3 4 5

_______________________

Please answer the following questions before you reach the end.

1. In which province do you live: _______

您所居住的省份是：________

1. In which city do you live: ______

您所居住的城市是：_______

1. What is your highest academic achievement? _____ (junior high/high school/community~~/~~ college/bachelor/master/doctorate)

您的最高学历是：______ (初中/高中/中专/大专/本科/研究生/博士)

1. In which province does your child take music lessons: _____

孩子进行音乐学习的省份是：______

1. In which city does your child take music lessons: _____

孩子进行音乐学习的城市是：______

1. At what age did your child begin taking private music lessons: ______

孩子几岁开始学习音乐：_______

1. How many years has your child been taking private music lessons? __________

孩子学习这门乐器多长时间了：_______

1. How long is each private music lessons: (15mins or less /30min/45mins/1hr/1 hour or more)?

孩子每节课的时长是：________ (15分钟或者少于15min/30分钟/45分钟/一小时或以上)

1. How frequently is the lesson: ______ (every week/every two weeks/every month/irregular)

孩子上课的周期是：______ (每周/每两周/每月/不固定时间)

1. How much does your child’s private music lesson cost per lesson: ______

每堂音乐课的费用是：______

1. How often do you pay for your child’s music lessons?

多久给孩子付一次学费：______ (每次/每周/每两周/每月/其它：____)

- 1. per lesson
  2. weekly
  3. every two weeks
  4. monthly
  5. other (please indicate here ________________)

# APPENDIX B: 80-ITEMS VERSION

**Demographic Information**

**基本信息调查：**

1. Are you a father or a mother: Father____ Mother______

您是孩子的父亲还是母亲: 父亲______ 母亲_______

1. How many children do you have? ______

您有几个孩子：______

1. How many of your children are taking music lessons? ______

您家里有几个孩子在进行课外音乐学习：________

Please select one child for the remainder of the survey if you have more than one child:

如果你有一个以上的孩子进行课外音乐学习，请选择其中一个孩子来完成以下调查：

1. Your child’s gender: Boy__ Girl___

孩子的性别是：男孩___ 女孩____

1. Your child’s age: ______

孩子的年龄是：_______

1. What is your child’s primary instrument? _______

孩子学习的主要乐器是什么：______

1. Besides the primary instrument, what other musical instrument is your child learning:______

除了以上乐器，孩子还学习其他乐器吗：________

**Please answer each one by rating yourself from 1-5, with 1 being totally disagree, to 5 totally agree.**

**请根据您的实际情况来对自己进行评估，“1”代表非常不同意，“5”代表非常同意。**

**1-Strongly disagree**

**非常不同意**

**2-Disagree**

**不同意**

**3- Neutral**

**中立**

**4- Agree**

**同意**

**5-Strongly agree**

**非常同意**

1. To support my child’s music learning, I buy him/her many musical books and magazines. (proactivity)

1.我购买很多音乐类书籍和杂志来支持孩子的音乐学习。

1 2 3 4 5

_______________________

1. To support my child’s music learning, I only buy him/her the needed musical score. (passivity)
2. 为了支持孩子的音乐学习，我只给孩子购买他需要的乐谱。

1 2 3 4 5

_______________________

1. I purchase an instrument for my child when the price is acceptable. (passivity)
2. 当价格可接受时，我才给孩子买乐器。

1 2 3 4 5

_______________________

1. To support my child’s music learning, I buy him/her more musical books and magazines only if needed. (passivity)
2. 我只购买需要的音乐类书籍和杂志来支持孩子的音乐学习。

1 2 3 4 5

_______________________

1. To support my child’s private music learning and provide a musical learning environment at home, I watch or listen to AV materials in the performance of the instrument with my child together only if needed. (passivity)
2. 为了支持孩子的音乐学习以及给他在家里提供一个良好的音乐氛围，我只在需要时才跟孩子一起听看音乐会视频。

1 2 3 4 5

_______________________

1. I enroll my child for a music level exam as other people’s children have enrolled in a music level exam. (passivity)
2. 我给我的孩子报名参加考级是因为别人的孩子都报名考级。

1 2 3 4 5

_______________________

1. If the teacher allows, I accompany my child during the private music lessons and observe. (proactivity)
2. 在孩子上音乐课的时候，如果老师允许的话，我陪在孩子旁边看他上课。

1 2 3 4 5

_______________________

1. My child does not have a designated area (e.g., for storing and practicing the instruments) as his/her musical space at home, but I am able to make it if there is extra space at home. (passivity)
2. 我孩子在家里没有固定的音乐区域（例如：练习乐器的地方，乐器摆放的地方），但是如果家里有多余的地方，我才会给孩子提供一个属于他的固定的音乐区域。

1 2 3 4 5

_______________________

1. I purchase other musical accessories, such as a metronome, for my child only if the teacher requires it. (passivity)
2. 我只给孩子购买老师要求的音乐学习辅助工具，例如：节拍器等。

1 2 3 4 5

_______________________

1. I chat with my child regarding music only when he/she shows the need for it. (passivity)
2. 当孩子有需要时，我才跟他聊音乐。

1 2 3 4 5

_______________________

1. I do not take him/her to any concert and public musical activities, even if it will help my child to continue his/her music learning experience (avoidance)
2. 就算对孩子未来音乐学习有帮助，我也不带他参加任何音乐会或者其他音乐活动。

1 2 3 4 5

_______________________

1. I do not accompany his/her practice in any circumstance. (avoidance)
2. 在任何情况下我都不陪伴孩子练习乐器。

1 2 3 4 5

_______________________

1. To support my child’s private music learning and provide a musical learning environment at home, I play AV materials in the performance of the instrument only if needed. (passivity)
2. 为了支持孩子的音乐学习以及给他在家里提供一个良好的音乐氛围，我只在需要时才在家里给孩子播放音乐会视频。

1 2 3 4 5

_______________________

1. During my child’s private music lessons, I wait outside. (passivity)
2. 在孩子上音乐课时，我在外面等。

1 2 3 4 5

_______________________

1. I purchase my child his/her own instrument to support his/her music practice. (proactivity)
2. 为了支持孩子学习音乐，我给他买属于他的乐器。

_______________________

1. I do not buy my child his/her own instrument. (avoidance)

16.我不给孩子买属于他的乐器。

1 2 3 4 5

_______________________

1. I enroll my child for an instrumental competition as other people’s children have enrolled in an instrumental competition. (passivity)

17.我给我的孩子报名参加器乐比赛是因为别人的孩子参加了乐器比赛。

1 2 3 4 5

_______________________

1. I do not chat with my child regarding music. (avoidance)
2. 我不跟孩子聊音乐。

1 2 3 4 5

_______________________

1. When I perceive that my child is gradually losing his/her interest in practicing music, I encourage and accompany her/him to keep up with their music practice. (proactivity)
2. 当我发现孩子对练习乐器逐渐失去兴趣时，我鼓励孩子并陪伴孩子一起练习。

1 2 3 4 5

_______________________

1. I do not enroll my child for an instrumental competition. (avoidance)
2. 我不给孩子报名参加任何器乐比赛。

1 2 3 4 5

_______________________

1. Regardless of my musical knowledge, I am willing to be part of my child’s music learning journey only if my child needs me. (passivity)
2. 无论我的音乐能力如何，我只有在孩子需要时才会愿意参与孩子的音乐学习。

1 2 3 4 5

_______________________

1. When the music teacher noticed that my child is very talented in a particular instrument and suggested learning from a more advanced music teacher, I take my child to the more advanced music teacher as long as it is easy to fit into our schedule (including transportation time). (passivity)
2. 当老师认为我的孩子在某个乐器方面很有天赋并建议孩子去跟更专业的老师学习时,如果时间或交通方便的话，我才送孩子去跟更专业的老师学习。

1 2 3 4 5

_______________________

1. When my child is tired of the instrument that he/she is currently learning, I shall see if there is another instrument that is available. (passivity)
2. 当我的孩子厌倦了他现在所学的乐器时，我才看看是否学习其他乐器。

1 2 3 4 5

_______________________

1. After each private music lesson, I do not encourage him/her. (avoidance)
2. 每次音乐小课后，我都不鼓励我的孩子。

1 2 3 4 5

_______________________

1. Regardless of my musical knowledge, I am willing to be part of my child’s music learning journey only if the teacher requires it. (passivity)
2. 无论我的音乐能力如何，我只在老师要求时才会愿意参与孩子的音乐学习。

1 2 3 4 5

_______________________

1. I purchase other musical accessories for my child, such as a metronome. (proactivity)
2. 我给孩子购买其他音乐学习辅助工具，例如：节拍器等。

1 2 3 4 5

_______________________

1. In a designated area (e.g., for storing and practicing the instruments) of our home, my child has his/her own musical space. (proactivity)
2. 我的孩子在家里有属于他自己固定的音乐区域（例如：练习乐器的地方，乐器摆放的地方）。

1 2 3 4 5

_______________________

1. Regardless of my musical knowledge, I am willing to be part of my child’s music learning journey. (proactivity)
2. 无论我的音乐能力如何，我都会积极参与并陪伴孩子进行音乐学习。

1 2 3 4 5

_______________________

1. To support my child to continue his/her music learning experience, I take my child to participate in more music concerts or public musical activities. (proactivity)
2. 为了支持孩子继续音乐学习，我带他参加音乐会及其他音乐活动。

1 2 3 4 5

_______________________

1. When I perceive that my child is gradually losing his/her interest in practicing music, I reduce the practice time. (passivity)
2. 当我发现孩子对练习乐器逐渐失去兴趣时，我减少孩子练习的次数。

1 2 3 4 5

_______________________

1. Before my child’s private music lessons, I drop my child off and leave. (avoidance)
2. 在孩子上音乐课之前，我把孩子送到上课地点就离开。

1 2 3 4 5

_______________________

1. I enroll my child for an instrumental competition as I foresee its benefits for my child. (proactivity)
2. 我给我孩子报名参加乐器比赛是因为对孩子有好处。

1 2 3 4 5

_______________________

1. I do not play AV materials in the performance of the instrument at home, even if it supports my child’s future private music learning and provides a musical learning environment at home. (avoidance)
2. 就算对孩子未来的音乐学习有帮助以及给他在家里提供一个良好的音乐氛围，我也不给孩子播放音乐会视频。

1 2 3 4 5

_______________________

1. Regardless of my musical knowledge, I am not willing to be part of my child’s music learning journey at all. (avoidance)
2. 无论我的音乐能力如何，我都不愿意参与孩子的音乐学习。

1 2 3 4 5

_______________________

1. After each private music lesson, I communicate with the music teacher about my child’s performance. (proactivity)
2. 每次音乐小课后，我都询问老师我孩子在课上的表现。

1 2 3 4 5

_______________________

1. After finishing each private music lesson, I reflect on it with my child together on what he/she has learned that day. (proactivity)
2. 在孩子上完每一堂音乐小课后，我跟孩子一起回顾当天在课上所学的内容。

1 2 3 4 5

_______________________

1. After each private music lesson, I encourage him/her. (proactivity)
2. 每次音乐小课后，我都鼓励我的孩子。

1 2 3 4 5

_______________________

1. When the music teacher noticed that my child is very talented in a particular instrument and suggested learning from a more advanced music teacher, I do not take my child to the more advanced music teacher. (avoidance)
2. 当老师认为我的孩子在某个乐器方面很有天赋并建议孩子去跟更专业的老师学习时,我也不送我的孩子去跟随更专业的老师学习。

1 2 3 4 5

_______________________

1. I sacrifice my free time to accompany my child in his/her instrumental practice. (Proactivity)
2. 我牺牲我的下班及休息时间陪伴孩子练习乐器。

1 2 3 4 5

_______________________

1. I do not purchase other musical accessories such as a metronome for my child. (avoidance)
2. 我没有给孩子购买其他音乐学习辅助工具，例如：节拍器等。

1 2 3 4 5

_______________________

1. Regardless of my child’s enjoyment in learning the instrument that he/she is currently learning, I decide what instrument he/she will continue to learn without communicating with my child. (avoidance)
2. 不管孩子是否喜欢他现在所学的乐器与否，我都不跟孩子沟通并自己为孩子继续学习音乐而做决定。

1 2 3 4 5

_______________________

1. I do not buy him or her other musical books or magazines even if it may support my child’s music learning. (avoidance)
2. 就算对孩子的音乐学习有帮助，我也不给孩子购买其他音乐类书籍或杂志。

1 2 3 4 5

_______________________

1. I listen to their practice while doing my daily chores and activities. (passivity)
2. 我一边做自己的事情，一边听着我孩子练习乐器。

1 2 3 4 5

_______________________

1. After each private music lesson, I communicate with the music teacher about my child’s performance only when the teacher contacts me. (passivity)
2. 每次音乐小课后，当老师先来跟我说，我才会跟老师交流我孩子在课上的表现。

1 2 3 4 5

_______________________

1. When I perceive that my child is gradually losing his/her interest in practicing music, I allow the child not to practice anymore. (avoidance)
2. 当我发现孩子对练习乐器逐渐失去兴趣时，我允许孩子不练习了。

1 2 3 4 5

____________________

1. During my child’s private music lessons, I take notes of the lesson only if the teacher requires it. (passivity)
2. 在孩子上音乐课的时候，如果老师要求，我才为他做笔记。

1 2 3 4 5

_______________________

1. After each private music lesson, I praise him/her. (proactivity)
2. 每次音乐小课后，我都表扬我的孩子。

1 2 3 4 5

_______________________

1. To support my child to continue his/her music learning experience, I take him/her to concerts or public musical activities only if the teacher requires it. (passivity)
2. 为了支持孩子继续音乐学习，如果老师要求，我才带他参加音乐会及其他音乐活动。

1 2 3 4 5

_______________________

1. I do not buy him/her musical score even if it may support my child’s music learning. (avoidance)
2. 就算对他的音乐学习有帮助我都不给孩子购买乐谱。

1 2 3 4 5

_______________________

1. My child does not have a designated area (e.g., for storing and practicing the instruments) as his/her musical space at home, but I am able to make it if his/her peers have it. (passivity)
2. 我孩子在家里没有固定的音乐区域（例如：练习乐器的地方，乐器摆放的地方），但是如果别的孩子都有自己固定的音乐区域，我才会为我的孩子提供。

1 2 3 4 5

_______________________

1. I do not watch or listen to AV materials in the performance of the instrument with my child, even if it supports my child’s future private music learning and provides a musical learning environment at home. (avoidance)
2. 就算对孩子未来的音乐学习有帮助以及给他在家里提供一个良好的音乐氛围, 我也不跟孩子一起听看音乐会视频。

1 2 3 4 5

_______________________

1. To support my child’s music learning, I buy him/her many musical scores. (proactivity)
2. 为了支持孩子的音乐学习，我给他购买很多的乐谱。

1 2 3 4 5

_______________________

1. I chat with my child regarding music often. (proactivity)
2. 我经常跟我的孩子聊音乐。

1 2 3 4 5

_______________________

1. To support my child’s private music learning and provide a musical learning environment at home, I play AV materials in the performance of the instrument. (proactivity)
2. 为了支持孩子的音乐学习以及给他在家里提供一个良好的音乐氛围，我在家播放音乐会视频。

1 2 3 4 5

_______________________

1. After finishing each private music lesson, I reflect on it only when the teacher requires me to do so. (passivity)
2. 在孩子上完每一堂音乐小课后，如果老师要求，我才跟孩子一起回顾当天课上所学的内容。

1 2 3 4 5

_______________________

1. Please choose number 2 below:

56.请在以下数字中选择数字2：

1 2 3 4 5

_______________________

1. During my child’s private music lessons, if the teacher allows, I use my phone to take video notes of my child (proactivity).

57.在孩子上音乐课的时候，如果老师允许的话，我通过手机录像来为他做视频笔记。

1 2 3 4 5

_______________________

1. My child does not have a designated area (e.g., for storing and practicing the instruments) as his/her musical space at home, and I am not able to make it for him/her. (Avoidance)

58.我孩子在家里没有固定的音乐区域（例如：练习乐器的地方，乐器摆放的地方），并且我也不会为孩子在家里提供一个属于他的固定的音乐区域。

1 2 3 4 5

_______________________

1. To support my child’s private music learning and provide a musical learning environment at home, I watch or listen to AV materials in the performance of the instrument with my child. (proactivity)
2. 为了支持孩子的音乐学习以及给他在家里提供一个良好的音乐氛围，我跟孩子一起听看音乐会视频。

1 2 3 4 5

_______________________

1. I send my child to take private music lessons because my friend’s or my neighbor’s children are taking music lessons.
2. 我送孩子去学音乐是因为我朋友或邻居的小孩都在学。

1 2 3 4 5

_______________________

1. I send my child to take private music lessons because I want my child to take music lessons.
2. 我送孩子去学音乐是因为是我希望我的小孩学。

1 2 3 4 5

_______________________

1. I send my child to take private music lessons because my child wants to take it.

62.我送孩子去学音乐是因为我的孩子想学。

1 2 3 4 5

_______________________

Please answer the following questions before you reach the end.

1. In which province do you live: _______

您所居住的省份是：________

1. In which city do you live: ______

您所居住的城市是：_______

1. What is your highest academic achievement? _____ (junior high/high school/community~~/~~ college/bachelor/master/doctorate)

您的最高学历是：______ (初中/高中/中专/大专/本科/研究生/博士)

1. In which province does your child take music lessons: _____

孩子进行音乐学习的省份是：______

1. In which city does your child take music lessons: _____

孩子进行音乐学习的城市是：______

1. At what age did your child begin taking private music lessons: ______

孩子几岁开始学习音乐：_______

1. How many years has your child been taking private music lessons? __________

孩子学习这门乐器多长时间了：_______

1. How long is each private music lessons: (15mins or less /30min/45mins/1hr/1 hour or more)?

孩子每节课的时长是：________ (15分钟或者少于15min/30分钟/45分钟/一小时或以上)

1. How frequently is the lesson: ______ (every week/every two weeks/every month/irregular)

孩子上课的周期是：______ (每周/每两周/每月/不固定时间)

1. How much does your child’s private music lesson cost per lesson: ______

每堂音乐课的费用是：______

1. How often do you pay for your child’s music lessons?

多久给孩子付一次学费：______ (每次/每周/每两周/每月/其它：____)

- 1. per lesson
  2. weekly
  3. every two weeks
  4. monthly
  5. other (please indicate here ________________)

# APPENDIX C: 25-ITEMS VERSION (FINAL VERSION)

**Demographic Information**

**基本信息调查：**

1. Are you a father or a mother: Father____ Mother______

您是孩子的父亲还是母亲: 父亲______ 母亲_______

1. How many children do you have? ______

您有几个孩子：______

1. How many of your children are taking music lessons? ______

您家里有几个孩子在进行课外音乐学习：________

Please select one child for the remainder of the survey if you have more than one child:

如果你有一个以上的孩子进行课外音乐学习，请选择其中一个孩子来完成以下调查：

1. Your child’s gender: Boy__ Girl___

孩子的性别是：男孩___ 女孩____

1. Your child’s age: ______

孩子的年龄是：_______

1. What is your child’s primary instrument? _______

孩子学习的主要乐器是什么：______

1. Besides the primary instrument, what other musical instrument is your child learning: ______

除了以上乐器，孩子还学习其他乐器吗：________

**Please answer each one by rating yourself from 1-5, with 1 being totally disagree, to 5 totally agree.**

**请根据您的实际情况来对自己进行评估，“1”代表非常不同意，“5”代表非常同意。**

**1-Strongly disagree**

**非常不同意**

**2-Disagree**

**不同意**

**3- Neutral**

**中立**

**4- Agree**

**同意**

**5-Strongly agree**

| Subscales | Items |
| --- | --- |
| Parents act in proactivity | 36. After each private music lesson, I encourage him/her.   1. After finishing each private music lesson, I reflect on it with my child together on what he/she has learned that day. 2. After each private music lesson, I communicate with the music teacher about my child’s performance. 3. I chat with my child regarding music often.   50. After each private music lesson, I praise him/her.   1. To support my child’s private music learning and provide a musical learning environment at home, I play AV materials in the performance of the instrument. 2. During my child’s private music lessons, if the teacher allows, I use my phone to take video notes of my child. 3. Regardless of my musical knowledge, I am willing to be part of my child’s music learning journey.   6. To support my child’s music learning, I buy him/her many musical books and magazines.   1. In a designated area (e.g., for storing and practicing the instruments) of our home, my child has his/her own musical space. 2. I enroll my child for an instrumental competition as I foresee its benefits for my child. |
| Parents act in passivity | 1. Regardless of my musical knowledge, I am willing to be part of my child’s music learning journey only if my child needs me. 2. I chat with my child regarding music only when he/she shows the need for it.   4. I enroll my child for a music level exam as other people’s children have enrolled in a music level exam.   1. To support my child’s private music learning and provide a musical learning environment at home, I play AV materials in the performance of the instrument only if needed. 2. Regardless of my musical knowledge, I am willing to be part of my child’s music learning journey only if the teacher requires it.   3. I purchase an instrument for my child when the price is acceptable.   1. When I perceive that my child is gradually losing his/her interest in practicing music, I reduce the practice time. 2. When my child is tired of the instrument that he/she is currently learning, I shall see if there is another instrument that is available. |

**非常同意**

| Parents act in avoidance | 1. I do not watch or listen to AV materials in the performance of the instrument with my child, even if it supports my child’s future private music learning and provides a musical learning environment at home. 2. My child does not have a designated area (e.g., for storing and practicing the instruments) as his/her musical space at home, and I am not able to make it for him/her. 3. I do not buy him/her musical score even if it may support my child’s music learning. 4. I do not play AV materials in the performance of the instrument at home, even if it supports my child’s future private music learning and provides a musical learning environment at home. 5. I do not purchase other musical accessories such as a metronome for my child. (avoidance) 6. When the music teacher noticed that my child is very talented in a particular instrument and suggested learning from a more advanced music teacher, I do not take my child to the more advanced music teacher. |
| --- | --- |
